# A Markov model for the temporal dynamics of balanced random networks of finite size

**DOI:** 10.3389/fncom.2014.00142

**Published:** 2014-12-03

**Authors:** Fereshteh Lagzi, Stefan Rotter

**Affiliations:** Bernstein Center Freiburg and Faculty of Biology, University of FreiburgFreiburg, Germany

**Keywords:** Markov process, self-consistent noise model, balanced random network, nonlinear dynamics, networks of finite size, Wilson-Cowan type model

## Abstract

The balanced state of recurrent networks of excitatory and inhibitory spiking neurons is characterized by fluctuations of population activity about an attractive fixed point. Numerical simulations show that these dynamics are essentially nonlinear, and the intrinsic noise (self-generated fluctuations) in networks of finite size is state-dependent. Therefore, stochastic differential equations with additive noise of fixed amplitude cannot provide an adequate description of the stochastic dynamics. The noise model should, rather, result from a self-consistent description of the network dynamics. Here, we consider a two-state Markovian neuron model, where spikes correspond to transitions from the active state to the refractory state. Excitatory and inhibitory input to this neuron affects the transition rates between the two states. The corresponding nonlinear dependencies can be identified directly from numerical simulations of networks of leaky integrate-and-fire neurons, discretized at a time resolution in the sub-millisecond range. Deterministic mean-field equations, and a noise component that depends on the dynamic state of the network, are obtained from this model. The resulting stochastic model reflects the behavior observed in numerical simulations quite well, irrespective of the size of the network. In particular, a strong temporal correlation between the two populations, a hallmark of the balanced state in random recurrent networks, are well represented by our model. Numerical simulations of such networks show that a log-normal distribution of short-term spike counts is a property of balanced random networks with fixed in-degree that has not been considered before, and our model shares this statistical property. Furthermore, the reconstruction of the flow from simulated time series suggests that the mean-field dynamics of finite-size networks are essentially of Wilson-Cowan type. We expect that this novel nonlinear stochastic model of the interaction between neuronal populations also opens new doors to analyze the joint dynamics of multiple interacting networks.

## 1. Introduction

Cortical neurons of behaving animals show highly irregular patterns of activity. One hypothesis for the source of such irregularity is the balance of excitation and inhibition in the steady state activity of the network (Softky and Koch, 1993; Bell et al., 1994; Shadlen and Newsome, 1994, 1998; Tsodyks and Sejnowski, 1995; van Vreeswijk and Sompolinsky, 1996). Experimental evidence in favor of this hypothesis suggest that excitation-inhibition balance is the principle of brain dynamics (Sanchez-Vives and McCormick, 2000; Shu et al., 2003; Haider et al., 2006; Okun and Lampl, 2008). The balanced state is an emergent self-consistent and stable solution of the temporal dynamics of the network (van Vreeswijk and Sompolinsky, 1996, 1998; Amit and Brunel, 1997b; Brunel, 2000). In other words, in a recurrent balanced network, both excitatory and inhibitory activity are shaped such that in cooperation with each other, they generate a stationary, self-consistent input-output behavior on the level of the mean and the fluctuations. The collective activity of the involved neuronal populations include weakly correlated and irregular spike trains. Due to its stochastic appearance, this feature is referred to as “self-generated noise” or simply “noise” in this paper. In fact, these fluctuations are generated mainly by the complex recurrent interactions in the network, even in absence of any external source of noise (van Vreeswijk and Sompolinsky, 1996; Kriener et al., 2008). As in our model there is no external source of noise, the fluctuations are very likely due to deterministic chaos in a high-dimensional system (for details see Jahnke et al., 2009).

Temporal fluctuations of neuronal activity reflect brain processes. Transient activity of neuronal networks, for instance, correspond to different neural computations at different stages of a cognitive task (see for example Churchland et al., 2011) or the representation of information in the brain (Destexhe and Contreras, 2006). Fluctuations also influence sensory perception in the case of ambiguous input. This phenomenon has been modeled by a multi-stable noise-driven dynamical system in which activity fluctuations cause transitions between meta-stable fixed points (Moreno-Bote et al., 2007; Deco and Romo, 2008). It was shown that dynamic noise in a neuronal network also gives rise to different dynamical states of the network. Both theoretical and simulation studies have addressed the role of noise for dynamic stability, or for the emergence of oscillations in network dynamics (Brunel and Hansel, 2006; Ghosh et al., 2008; Touboul et al., 2011; Cai et al., 2012) which may have implications for brain functions. Therefore, to understand the functional properties of neuronal networks, it is essential to understand the dynamics of the time dependent variability in such systems. Theoretical studies of balanced random networks indicate that the fluctuations are essentially determined by two factors: neuronal correlations and the finite size of the network (Ginzburg and Sompolinsky, 1994; Brunel and Hakim, 1999; Brunel, 2000).

To address the fluctuations of the activity of a complex high-dimensional system such as a spiking neural network, a reduced low-dimensional description of network activity is needed. However, to compensate for the loss of degrees of freedom, an analytical treatment is needed such that the essential properties of the stochastic dynamics of the system fluctuations are preserved to an acceptable degree. Finding a suitable stochastic model to replace the spiking dynamics of a network is a challenge. One reason is that such networks are hybrid systems, as the membrane potential of each neuron is a continuous variable, and the spiking activities are discrete quantities. Secondly, an appropriate time scale has to be defined because the amplitude and the dynamics of the fluctuations depend on the temporal resolution. Thirdly, the stationary activity of the reduced model should maintain the statistical properties of the population activities, as their statistics are crucial for the switching dynamics of a network with two or more interacting populations (Bressloff and Newby, 2013). Finally, each neuron is a highly nonlinear element in a random network, due to its threshold and refractoriness. It has been hypothesized that in the thermodynamic limit, when the number of neurons becomes very large, the global dynamics is linearized due to the negative feedback from the inhibitory population (van Vreeswijk and Sompolinsky, 1996, 1998; Tetzlaff et al., 2012; Helias et al., 2013). However, in networks of finite size, as we show in this paper, the dynamics are not fully described by a linear framework.

An influential study on population dynamics of excitatory and inhibitory neurons has been performed more than 40 years ago (Wilson and Cowan, 1972). The authors considered an infinitely large number of neurons in each population such that a fraction of all neurons in the population are in the refractory state. They derived an *ad hoc* response function for the non-refractory neurons and using coarse-graining of activities in time, they derived a set of coupled ordinary differential equations. However, correlations and finite size fluctuations were not captured by this model. In essence, without fluctuations in the input, there are no output fluctuations in this model. In fact, most theoretical studies of population interactions using a mean-field approach (see e.g., Gerstner, 1995; Amit and Brunel, 1997a,b; Brunel, 2000; Aviel and Gerstner, 2006; Kriener et al., 2008; Toyoizumi et al., 2009; Cardanobile and Rotter, 2011; Ledoux and Brunel, 2011; Ostojic and Brunel, 2011) have not addressed the consequences of the finite size of the network and/or pairwise correlations for the temporal dynamics of the population activities in a self-consistent way.

There are a few explicit or implicit suggested approaches to study finite size population dynamics. The first type of studies are based on deterministic equations using mean-field approaches that are derived by an external source of noise (Kriener et al., 2008; Toyoizumi et al., 2009; Tetzlaff et al., 2012). Seminal studies on balanced random networks show that a stochastic input is not needed for network fluctuations and the noise in the system is self-generated as a result of recurrent activity and stochastic spiking of neurons (van Vreeswijk and Sompolinsky, 1996, 1998). Therefore, this approach cannot describe the temporal dynamics in a self-consistent way. Moreover, a deterministic set of equations with additive noise (Kriener et al., 2008) cannot reproduce the state dependent fluctuations of the network activity, as we will show in our study.

A second class of studies considered independent neurons with Poisson statistics (Brunel and Hakim, 1999; El Boustani and Destexhe, 2009). This approach could lead to unrealistic number of spike counts in a short time bin. Moreover, with the assumption of uncorrelated neurons, neural Poisson statistics result in network Poisson statistics. As we show in this article, the statistics of the population activities in balanced random networks are not Poissonian. Exploiting a general escape noise model in a point process framework, Spiridon and Gerstner (1999) derived an integral equation for the population activity of a fully coupled network. According to their approach, the finite size effect would show up as a multiplicative noise term in the original equation. As we show later, our analysis supports these results to some extent.

A third approach tries to describe either the dynamics of each neuron (Ohira and Cowan, 1993, 1995; Soula and Chow, 2007; El Boustani and Destexhe, 2009) or the network (Touboul and Ermentrout, 2011; Buice and Chow, 2013a) by a Markov process. The latter has the problem that possible jumps of the Markov process are limited to the immediate neighbors of each state; meaning that the number of active neurons in each population can either increase or decrease by one. The former approach seems to be able to better capture the dynamics and statistics of the network. Soula and Chow (2007) assumed a Markov model in discrete time with a time scale in the range of membrane time constant in the case of instantaneous synapses. The transition rate of a typical neuron in the network is calculated from the stationary firing rate and includes the net amount of excitation in the system. However, balanced random networks operate on a much faster time scale compared to that of a single neuron. Also, the interaction between excitation and inhibition, and the consequences of the negative feedback on temporal dynamics are not analyzed in this study. In another study, following a similar approach in continuous time, a master equation for the activity of a balanced network with current-based and conductance-based synapses was derived (El Boustani and Destexhe, 2009). This method needs the static transfer function of a single neuron that maps input rates to output rates.

Finally, population density approaches based on a conservation law imposed on the probability flux (the number of neurons is constant) (for details see Knight, 1972; Abbott and van Vreeswijk, 1993; Treves, 1993; Knight et al., 1996; Omurtag et al., 2000; Sirovich et al., 2000; Haskell et al., 2001; Nykamp and Tranchina, 2001; Mattia and Del Giudice, 2002) are yet another way of deriving stochastic dynamics from deterministic equations. In a study on the temporal dynamics of the interaction between excitation and inhibition in networks of finite size, an eigenfunction expansion of the essentially nonlinear Fokker-Planck equation resulted in a set of coupled ordinary differential equations(Mattia and Del Giudice, 2002, 2004). However, the noise term included in the model to account for the finite size of the network, was a white noise of fixed amplitude. Recently, Buice and Chow (2013a,b), using a population density method and moment hierarchies of the equation, derived path integrals of a moment generating functional. To get a time dependent correlation function of the system, by introducing a small perturbation, they applied a linear expansion of the equation of moments (Buice and Chow, 2013a,b).

In this paper, we aim at describing statistics and dynamics of finite-size fluctuations in a balanced random network of excitatory and inhibitory neurons self-consistently, such that the temporal dynamics of the network is driven by the interactions in the network. Moreover, a high correlation between the excitatory and inhibitory population, in their stochastic representation in the model, has to be preserved. To this end, a typical neuron in the network is modeled by a two-state Markov system that its transition probabilities needs to be derived dependent on the network activity states. Our model is based on the state space analysis of numerical simulations of interactions between the excitatory and the inhibitory population in a large balanced network, in the regime dominated by inhibition.

Systematic analysis of the two-dimensional population spike counts shows that no dynamic model with additive Gaussian noise can fully describe the temporal dynamics of the network activity. Specifically, the more excitation and the less inhibition is recruited in the network at any given point in time, the higher is the variance of the self-generated noise. It will be demonstrated in this paper that a stationary external input results in nonlinear interactions between excitation and inhibition. Moreover, we will show that the self-generated and state dependent noise emerges naturally as a result of the finite number of neurons in the network. Furthermore, the suggested two-state Markov model is capable of producing a heavy-tailed (positively skewed) distribution of excitatory and inhibitory spike counts, a property of balanced random networks that is considered here for the first time. We show that this heavy-tailed distribution can be well approximated by a log-normal distribution.

## 2. Materials and methods

In this section, we first describe our assumptions about the structure and the parameters of the network. Then, we show how to reconstruct the dynamic flow based on the results of spiking network simulations. In Section “Estimation of the dynamic flow underlying the mean-field dynamics” we first suggest a Markov model to represent the collective activity of neuronal populations, with transition probabilities inferred from numerically simulated networks of spiking neurons. The objective of this study is to identify a suitable bin size to represent the temporal dynamics of the network fluctuations. Then, in Section “Markov model for the mean-field and stochastic dynamics” we introduce a two-state Markov model for each neuron, termed the “Active-Refractory Markov” (ARM) model. A method to find the mean-field equations and a self-consistent noise model based on the Markovian single-neuron dynamics is introduced at the end of this section.

### 2.1. Network structure and parameters

The network under study is composed of 10,000 excitatory and 2500 inhibitory neurons, similar to the network studied by Brunel (2000). Each neuron receives local inputs from randomly chosen fraction of the excitatory and the inhibitory population (10% each). An external Poisson process of rate 25 spikes/ms mimics the input from other brain areas. The neurons are modeled by a leaky integrate-and-fire (LIF) dynamics with pulse-like post-synaptic currents (PSC), and exponential post-synaptic potentials (PSP). Therefore, the dynamics of neuron *i* in the network, regardless of whether it is excitatory or inhibitory, satisfies

(1) $$\tau {\dot{v}}_{i}(t)=-v_{i}(t)+\tau \sum_{j=1}^{N}J_{ij}S_{j}(t-t_{d})$$

where *S_j_*(*t*) = ∑_*k*_δ(*t* − *t^j^_k_*) is the spike train of neuron *j*, which is seen by the postsynaptic neuron with a delay of *t_d_*, and integrated by it with a membrane time constant τ = 20 ms. *J*_*ij*_ is the amplitude of the post synaptic potential (PSP), *J* = 0.1 mV for the available excitatory synapses to each neuron and −*gJ* = −0.6 mV for inhibitory synapses. *N* is the total number of neurons in the network. The membrane potential of each neuron, once reached the threshold at θ = 20 mV, is reset to *v*_reset_ = 10 mV and a spike will be generated. The membrane potential remains at *v*_reset_ for a refractory period of 2 ms. These parameters are identical to those studied in Brunel (2000) and represent a simplified model for one cubic millimeter of neocortical tissue. The results of our study are valid for a wide range of biologically realistic parameters, as far as the network is in the inhibition dominated regime. Numerical simulations of the network were all conducted in NEST (Gewaltig and Diesmann, 2007) with a time resolution of 0.01 ms and a minimal synaptic delay *t_d_* equal to the time resolution. The network simulation time was 100 s. As a result, a total number of 10^7^ data points for each population in a histogram with a bin size of 0.01 ms was available for further data analysis.

### 2.2. Estimation of the dynamic flow underlying the mean-field dynamics

We reconstructed the flow corresponding to the mean-field dynamics from a simulated time series of the spiking network activities. To this end, for each possible combination (*i, j*) of excitatory spike count *i*, and inhibitory spike count *j*, the “state” of the system, we first collected the corresponding derivatives by computing the increments in successive time bins divided by the time bin, for both excitatory and the inhibitory spike counts. Taking the mean of the encountered derivatives for each state gave the average direction into which the system moved forward in time. This way, for each state visited in the simulation, a velocity vector is obtained, yielding a vector field that approximates the flow in a two-dimensional state space. Calculating the variance of the state dependent derivatives results in the state dependent variance of the self generated noise (fluctuations) in the system (see Results for more details).

### 2.3. Markov model for the mean field and stochastic dynamics

As mentioned in the beginning of this section, we considered two different Markov models for different purposes. First, for the selected time bin *dt*, chosen such that a Markov model is capable of reproducing the power spectrum of the network activity with the highest fidelity, a state transition matrix was inferred entirely based on the data obtained from a spiking network simulation. It is important to stress that the Markov model for the selected bin size *dt* provides a compressed description of the network dynamics, assuming that only the most recent bin, and no longer account of the history of the network activity, determines the dynamics of the network at any given point in time. The analysis described here explores the limits of such a description of the the large-scale dynamics, and in this paper we refer to it as “Markov chain analysis.” However, deriving the transition matrix from a self-consistent analysis is difficult. Therefore, we looked for a more analytically tractable approach in a second step. This approach, which is the main focus of this study, is based on a two-state Markov description of individual neurons. As shown in this article, this model is able to describe both the dynamics and the statistics of the collective activity of the network in a self-consistent way. The transition rates of this model are also estimated form the spiking network simulations.

#### 2.3.1. Markov chain analysis

**Figure 1:**
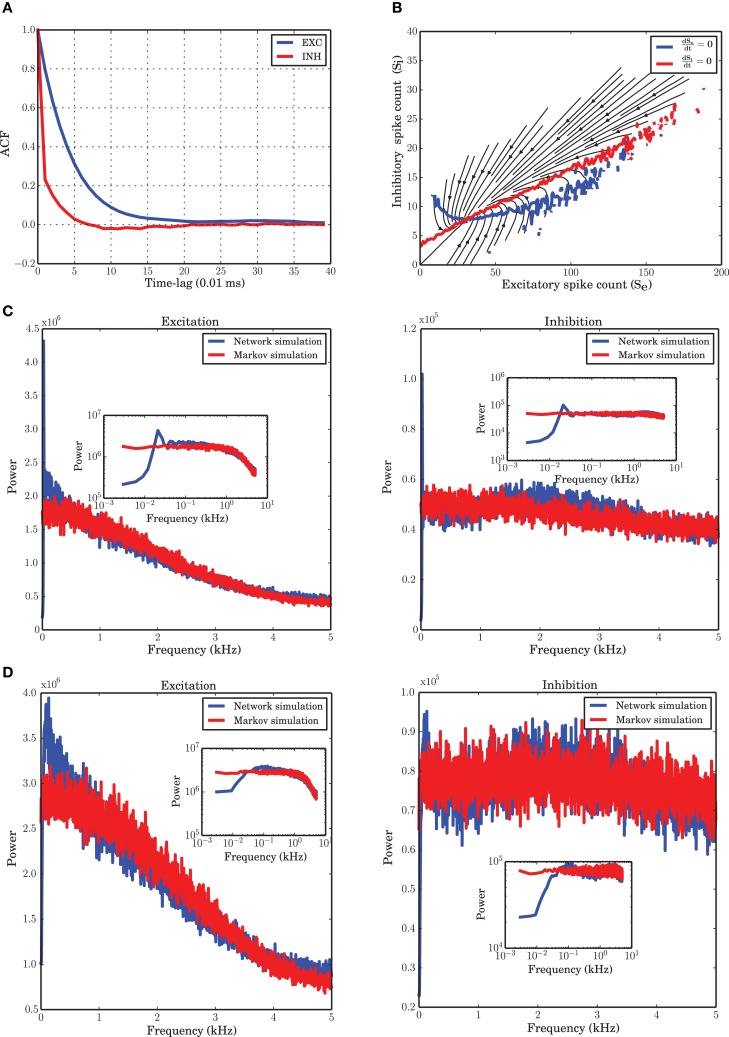
**Markov model simulation compared with a spiking neuronal (SNN) simulation. (A)** Autocorrelation function of the excitatory and inhibitory populations with a temporal resolution of 0.01 ms. Inhibition shows faster dynamics and shorter memory. **(B)** Vector field of one realization of the Markov chain with all probabilities for state transitions inferred from the original simulation of the spiking network. **(C)** Power spectrum of the excitatory and inhibitory population signal (PSTH) of SNN (blue curves), and generated by a simulation of the Markov model (red curves) corresponding to a spiking network simulation with *v*_reset_ = 0 mV. The low frequency dynamics are not very well captured by the Markov model; however, the overall shape of the spectrum, as well as the frequency beyond which the power drops, are well preserved. There are several peaks in the low frequency regime, corresponding to slow network oscillations. **(D)** Power spectral density for a simulation with *v*_reset_ = 10 mV. In contrast to the case of stronger reset, **(C)**, there are fewer peaks in the low frequency range.

In this section we address the question whether we can identify a time scale such that a Markov model can describe the time dependent dynamics of the network sufficiently well. To that end, based on the joint time series of the two population spike counts, a matrix of transition probabilities for any possible jump from state (*i, j*) at time *t* to state (*i*′, *j*′) at time *t* + *dt* was inferred. Starting from an arbitrary initial condition, a stochastic signal was then generated which was able to match the dynamics and statistics of the two populations PSTH sufficiently well (Figures 1B–D). However, as the power spectra of the stochastic implementation of the Markov model were not exactly identical to those of the spiking network simulations, even for the optimal step size *dt* (see next subsection), we conclude that the Markov property is only approximately satisfied. This approximation to the complicated dynamics of the system provides a simple model for the stochastic behavior of the network. It is important to check which properties of the system can be explained by a Markov model of the type discussed here. Some limits of the model due to this assumption are described in the results and will be further analyzed in the Discussion Section.

#### 2.3.2. Selection of the bin size

To extract the activity of the two neuronal populations, we consider a variant of the Peri-Stimulus Time Histogram (PSTH) of the spike trains of the excitatory and inhibitory neuronal populations. To find a suitable time resolution such that the dependency of the dynamics on the past history is essentially reduced to the most recent time bin, we looked at the autocorrelation functions of the PSTH of each population. As commonly done, we considered the first zero-crossing of the autocorrelation function as a first estimate for the time scale of the dynamics. As Figure 1A shows, however, the two populations have slightly deviating time scales. In the range of these two time scales, we explored a set of bin sizes that were integer multiples of the temporal resolution for the spiking network simulations (0.01 ms). For each value, we reconstructed the transition probabilities from the data, as explained in Section “Markov chain analysis.” Using these transition probabilities, a stochastic signal for both populations was generated and the power spectra of the signals were compared with those of the spiking network simulations extracted at the same bin size. We found that a bin size of 0.1 ms provides power spectral densities best matching to those obtained from spiking network simulations for both populations (Figure 1D). This yields a reasonable time basis for the dynamics, based on numerical experiments, in line with the Markov assumption. Due to the discrete nature of spike counts in each bin, the dynamics in this paper is analyzed in discrete time, with a discrete noise model corresponding to the finite size of both neuronal populations.

#### 2.3.3. Two state active-refractory Markov model

**Figure 2:**
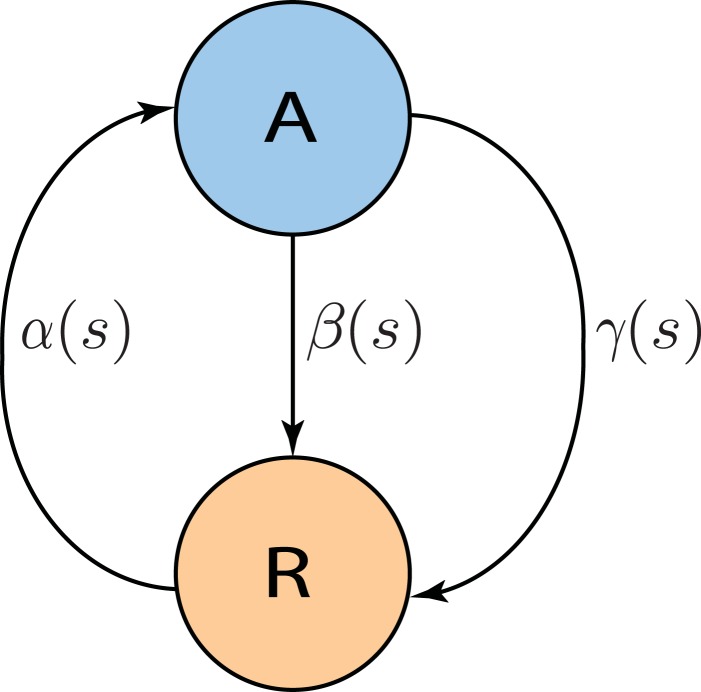
**Two state Active-Refractory Markov (ARM) model**. The membrane potential of such a neuron is either close to threshold [active state (A)] or far from threshold [refractory state (R)]. Spikes are generated by transitions in the γ branch. β is mainly determined by the leak in the membrane potential, and α causes transitions from the refractory to the active state. In general, the transition rates could be functions of spike counts; however, in the ARM model in this study, α is an exponential function; β and γ are constants. Neurons are independent of each other; therefore, the noise model has a binomial distribution, and is state dependent.

The goal is to derive a Markov model the parameters of which can be interpreted in terms of neural dynamics, and that captures the finite size effects. The model should also capture the properties of the spiking network simulation on the level of mean-field as well as the transient fluctuations. We came up with a two-state Markov model, where each neuron is assumed to generate its spikes independently of the other neurons, given the input from the rest of the network. Moreover, it is assumed that each neuron's membrane potential falls into one of two classes: close to threshold (active state) or far away from threshold (refractory state). Hence, we call this process the “Active-Refractory Markov” (ARM) model. A schematic of the neuron model is depicted in Figure 2. Transitions from the active to the refractory state can be of two different types. Either the membrane potential of a neuron decays due to the membrane leak [Equation (1)], or the neuron receives inhibitory input spikes, or the neuron fires a spike itself and the membrane potential is reset. The former is described by the β branch; the latter is due to the γ branch in the model (Figure 2). More specifically:

- α is the rate of transition from refractory to active state.
- β is the rate of transition from active to refractory state without any spike emission.
- γ is the rate of transition from active to refractory state due to spike generation and the reset afterwards.

In general, α, β, and γ are nonlinear functions of the state (spike counts). In our model, it turns out that α is an exponential function of a linear combination of spike counts; β and γ are constants estimated from the data. Note that the definition of a state in the ARM model deviates from the definition in the Markov chain model described in the previous section. In the latter, states are just spike counts, and transition probabilities are also defined in terms of spike counts. In the ARM model, however, the actual states are the occupation numbers of the active and refractory pools. These can neither be directly observed nor inferred from numerical simulations of the spiking neuronal network (SNN), but for our analysis we found a way work around this problem. All neurons in the network under study have the same in-degree and, consequently, share the same input statistics. Therefore, the transition rates are assumed to be identical for excitatory and inhibitory neurons. Neurons are assumed to make their state transitions, in particular spike firing, independently of each other, given their inputs.

The network is comprised of an excitatory and an inhibitory population. A population of size *N* will have *A* neurons in the active state and *N* − *A* neurons in the refractory state. Considering identical and independent transition rates for all neurons, our approach was to take neurons out of a state based on binomial distributions with the number of neurons in each state corresponding to the occupation numbers. The transition probability is given by multiplying the transition rate with *dt*, assuming that this is a small number. This results in the following stochastic description of the system

(2) $$\Delta A(t)=A(t+dt)-A(t)=\Delta A^{+}(t)-\Delta A^{-}(t)$$

In equation (2), Δ*A*^+^(*t*) and Δ*A*^−^(*t*) are the increment and decrement from the active pool *A* that indicate the number of incoming neurons from the refractory pool, and the number of outgoing neurons to the refractory pool, respectively. Δ*A*^−^(*t*) has a binomial distribution with parameters *A*(*t*) and (β + γ)*dt* as the number of available neurons and the probability of selection, respectively. Therefore,

(3) $$p\left(\Delta A^{-}(t)=x\right)=\left(\begin{array}{c} A(t)\\ x \end{array}\right){\left((\beta +\gamma )dt\right)}^{x}{\left(1-(\beta +\gamma )dt\right)}^{A(t)-x}$$

where $\left(\begin{array}{c} A(t)\\ x \end{array}\right)=C_{A(t)}^{x}$ is the binomial coefficient. In this model, the spike count *S* is determined by the number of neurons in the active state and the transition rate γ in two steps: First, the total number of outgoing neurons from the active pool is calculated from a binomial distribution with probability (β + γ)*dt*. This quantity is exactly equal to Δ*A*^−^(*t*). Second, from Δ*A*^−^(*t*), the neurons that are actually firing a spike are drawn with another binomial distribution with probability $\frac{\gamma }{\gamma +\beta }$. Therefore,

(4) $$p\left(S(t)=z\right)=C_{\Delta A^{-}(t)}^{z}{\left(\frac{\gamma }{\gamma +\beta }\right)}^{z}{\left(\frac{\beta }{\gamma +\beta }\right)}^{\Delta A^{-}(t)-z}$$

A similar expression as the one given in equation (3), a binomial distribution with rate α, describes the number of incoming neurons to the active pool at time *t*.

(5) $$p\left(\Delta A^{+}(t)=y\right)=C_{N-A(t)}^{y}{\left(\alpha dt\right)}^{y}{\left(1-\alpha dt\right)}^{N-A(t)-y}$$

With *A* neurons in the active state and *N* − *A* neurons in the refractory state at time *t*, the dynamic equation describing the expected value of the dynamics of *A* is

(6) $$\frac{d}{dt}E[A(t)]=\alpha (N-E[A(t)])-(\beta +\gamma )E[A(t)]$$

It is important to point out that the number of neurons in the active state, *A*(*t*), is not observable, as we record only spikes. To describe the dynamics of the network it is, therefore, easier to describe the temporal dynamics in terms of observables, like the number of spiking neurons *S*(*t*). As mentioned before, *S* is the integer number of spikes generated in the γ branch, where 𝔼[*S*] = 𝔼[*A*]γ*dt*. It is straightforward to rewrite equation (6) as a function of *S* and get an equation describing the temporal dynamics of the spike counts

(7) $$\frac{d}{dt}E[S(t)]=\alpha (N\gamma dt-E[S(t)])-(\beta +\gamma )E[S(t)]$$

For better readability, we will drop the 𝔼[.] operator that indicates the expected values of *A* and *S*. As all neurons are assumed to have the same membrane potential dynamics, determined by equation (1), and receive the same number of inputs regardless of their identity, the two populations have identical transition rates and therefore equation (7) holds for both populations. The only difference between the two populations, however, is the total number of neurons included in each of them. We denote the number of excitatory neurons by *N_e_* and the number of inhibitory neurons by *N_i_*. Excitatory and inhibitory spike counts generated at time *t* are given by *S_e_*(*t*) and *S_i_*(*t*), respectively. A nonlinear regression analysis of equation (7) applied to data from a spiking network simulation shows that α is an exponential function of recent spike counts, and that β and γ are well approximated by constant rates (see the Result Section for more details). Therefore, the two-dimensional mean-field has the following dynamics

(8) $$\{\begin{array}{l} \begin{array}{l} {\dot{S}}_{e}(t)=\exp\left(c_{0}+c_{1}S_{e}(t)+c_{2}S_{i}(t)\right)\left(N_{e}\gamma dt-S_{e}(t)\right)\\ \;-(\beta +\gamma )S_{e}(t)\\ {\dot{S}}_{i}(t)=\exp\left(c_{0}+c_{1}S_{e}(t)+c_{2}S_{i}(t)\right)\left(N_{i}\gamma dt-S_{i}(t)\right)\\ \;-(\beta +\gamma )S_{i}(t) \end{array} \end{array}$$

All the unknown parameters of the ODE system (8) can be estimated from the vector field extracted from simulated data. For this purpose, the regression analysis was performed with Python, using general purpose least-square optimization available in the SciPy library (Jones et al., 2001).

The exponential shape of α can be qualitatively justified. As pointed out in previous studies (Brunel and Hakim, 1999; Ricciardi et al., 1999; Brunel, 2000), the time dependent distribution of the membrane potential of a typical integrate and fire neuron, under the assumption of stochastic input and small PSPs, follows the Fokker-Planck equation with a drift and a diffusion term. In general, these two terms are functions of the recurrent activity of the network and therefore result in a nonlinear partial differential equation. However, under the assumption of Gaussian white noise input that is independent of the activity of the network, the steady state solution of equation (9), with appropriate boundary conditions, characterizes the stationary distribution of the membrane potential of a typical leaky integrate-and-fire (LIF) neuron. The dynamics of the distribution of the membrane potential is therefore

(9) $$\begin{array}{l} \tau \frac{\partial }{\partial t}p(v,t)=-\frac{\partial }{\partial v}\left[\left(-v+\tau \sum_{k}r_{k}(t)J_{k}\right)p(v,t)\right]\\ \;+\frac{1}{2}\left[\tau \sum_{k}r_{k}(t)J_{k}^{2}\right]\frac{\partial^{2}}{\partial v^{2}}p(v,t)\\ \;=-\frac{\partial }{\partial v}\left[\left(-v+\mu )p(v,t\right)\right]+\frac{\sigma^{2}}{2}\frac{\partial^{2}}{\partial v^{2}}p(v,t) \end{array}$$

where *r_k_*(*t*) is the firing rate of the pre-synaptic source *k* and *J_k_* is the corresponding PSP to source *k*. μ and σ are the first and second moments, respectively, of the external input to the neuron. Equation (9) is based on the conservation of the probability flux of the membrane potential and could be rewritten in the following form:

(10) $$\tau \frac{\partial }{\partial t}p(v,t)=-\frac{\partial }{\partial v}\Phi (v,t),$$

where Φ(*v, t*) represents the probability flux and shows the probability mass crossing any arbitrary *v* per unit of time *t*. The stationary time independent solution of *p*(*v*) without considering refractoriness and with appropriate boundary conditions at threshold θ, *v*_reset_ and *v* = −∞ is (Gerstner and Kistler, 2002)

(11) $$p(v)=\{\begin{array}{l} \begin{array}{l} \frac{c_{1}}{\sigma }\exp\left(-\frac{{\left(v-\mu \right)}^{2}}{\sigma^{2}}\right)\;v<v_{\text{reset}}\\ \frac{c_{2}}{\sigma }\exp\left(-\frac{{\left(v-\mu \right)}^{2}}{\sigma^{2}}\right)\int_{v}^{\theta }\exp\frac{{\left(\text{x-}\mu \right)}^{\text{2}}}{\sigma^{\text{2}}}\text{d}x\;v_{\text{reset}}<v<\theta \end{array} \end{array}$$

Intuitively, active and refractory states of a neuron are linked to high and low membrane potentials, respectively. The probability to encounter a potential exceeding a certain value, therefore, should be related to the rate α that describes a transition from refractory to active in the ARM model. The transition rate α, therefore, is proportional to the time dependent flux of the membrane potential crossing the border between the active and the refractory state. For simplicity we can approximate the time (state) dependent flux with the steady state flux plus some fluctuations. These fluctuations are determined by the spike counts of the activity: the larger the excitatory spike counts and the smaller the inhibitory spike counts are, the larger the probability flux crossing the border between the active and the refractory state is. As in equation (10), Φ is related to the voltage integral of *p*(*v, t*), the time dependent flux will be proportional to the local behavior of the Cumulative Density Function (CDF) of the membrane potential distribution. In our Result section, it is argued that for a wide range of the membrane potential between *v*_reset_ and θ, the stationary Cumulative Density Function (CDF) is locally well approximated by an exponential function, matching the functions we saw in numerical simulations of SNNs based on the LIF neuron model. A formal derivation of the link between the LIF and the ARM model, however, is mathematically involved and beyond the scope of the present paper.

## 3. Results

In this section, numerical results from the simulation of large but finite balanced random networks are illustrated, which all imply either the nonlinear dynamics of interactions, or the non-Gaussian and state dependent nature of the self-generated noise. A comparison between the spiking network simulation results and simulation of the Active-Refractory Markov (ARM) model is made to show the limits and strengths of the model.

### 3.1. Markov chain inferred from time series

A numerical study of the network shows that with a time bin of 0.1 ms the most essential features of the system under study are recovered by a Markov chain model where the probabilities for state transitions are all extracted from the data. The vector field obtained from the spike counts (Figure 1B) and the power spectral density of both populations modeled with a Markov chain (Figure 1D) are similar to those obtained from the simulation of the spiking network.

The autocorrelation function (ACF) of the excitatory and inhibitory spike counts (Figure 1A) reflect the memory of the system. It has been reported previously (Tetzlaff et al., 2012), and is confirmed again in our study, that the time constant of the decay is smaller for the inhibitory population, although the input to excitatory and inhibitory neurons are statistically the same. This behavior was hypothesized to be related to the negative feedback contribution of the inhibitory population in the large-scale dynamics of the network (Tetzlaff et al., 2012). To examine the Markovian nature of the dynamics, a good choice for the time bin of the histograms is the point where the autocorrelation has its first zero-crossing. For a simulation with a temporal resolution of 0.01 ms, this point is roughly 0.07 ms for the inhibitory population and 0.20 ms for the excitatory population, according to Figure 1A. Since a unique time scale for the model and simulations is needed, we chose 0.1 ms to construct the PSTH of both populations.

The low frequency power spectra of the populations are not very well captured by the Markov model. This may be caused by the fact that there is a dependence on the past spiking activity of the two populations, which cannot be reflected by the Markov process employed here. We wanted to find out which features of the system are nevertheless recoverable by a Markov process. The low frequency behavior of the system depends on the distance between *v*_reset_ and the spiking threshold θ (Figures 1C,D). The bigger this distance is, the more peaks in the low frequency part of the system appear. Refractoriness can also change the shape of the power spectrum in these frequency ranges (Franklin and Bair, 1995; Mar et al., 1999; Spiridon and Gerstner, 1999). It is obvious that a Markov process due to its lack of memory cannot capture this phenomenon.

### 3.2. Transition probabilities of the active-refractory Markov (ARM) model

**Figure 3:**
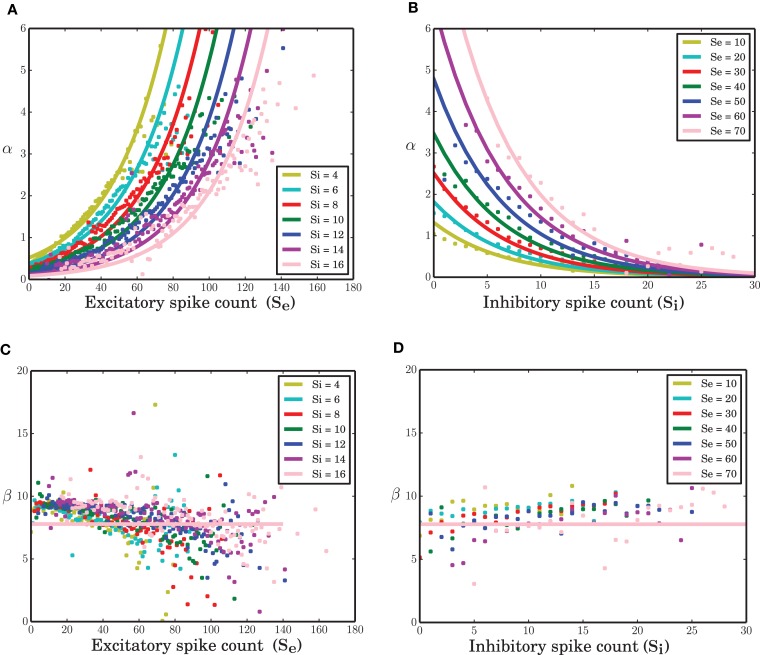
**Transition probabilities of the ARM model. (A,B)** α as a function of excitatory and inhibitory spike counts (dots) and an exponential fit to the data (solid lines). **(C,D)** β as a function of excitatory and inhibitory spike counts (dots) and a constant function (solid lines) fitted to the data.

All neurons in the network under study are statistically the same. Therefore, identical transition probabilities α, β, and γ were imposed for excitatory and inhibitory neurons. A nonlinear regression to estimate the parameters of equation (8) given the excitatory and inhibitory spike counts and their temporal derivatives from the time series results in an exponential link function for α. The function β exhibits a slight negative dependency on the excitatory spike counts (Figure 3C-dots). Ignoring this does not visibly affect the simulation results of the model (data not shown). Therefore, it was assumed to be a constant parameter. An exponential function for α and constant parameters for β (solid lines in Figure 3) and γ result in *c*_0_ = −0.046, *c*_1_ = 0.032, *c*_2_ = −0.152, β = 7.78 and γ = 0.325 (all quantities in spikes/ms) as the estimated parameters of the model in equation (8). Interestingly, *c*_2_, the coefficient of the inhibitory spike counts is roughly 5 times bigger in amplitude compared with *c*_1_. This was expected since IPSP = −*g* EPSP. In the stochastic implementation of the ARM model, the transition rate α is a function of random variables *S_e_* and *S_i_*. Therefore, the ARM model can be interpreted as a type of “doubly stochastic” point process.

**Figure 4:**
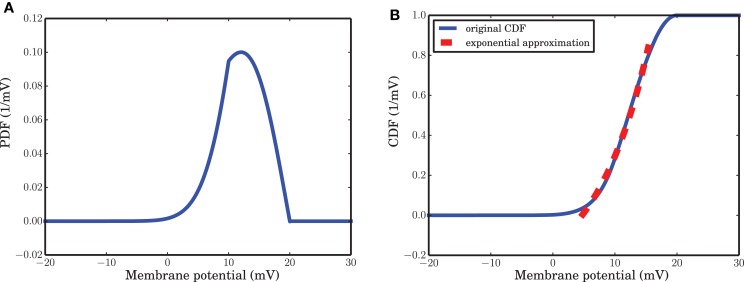
**Probability Density Function (PDF) and its cumulative give a hint for state probability transition α. (A)** Stationary PDF of the membrane potential of a leaky integrate and fire neuron. The value of the membrane potential that separates the active from the refractory state is somewhere between *v*_reset_ = 10 mV and θ = 20 mV. **(B)** Cumulative Density Function (CDF) of the stationary distribution of the membrane potential. For a wide range of membrane potential values the CDF is well approximated by an exponential function of *v*.

As mentioned in the Methods Section, for a wide range of membrane potentials *v* the CDF of the membrane potential can be approximated by an exponential function (Figure 4B). For values of the membrane potential close to threshold, however, the CDF does not match the fitted exponential function very well. This sub-exponential behavior is also observed in the data (dots in Figures 3A,B). This similarity suggests that a better approximation for α might come from the integral of the analytical solution of equation (9), but a formal mathematical analysis of this idea is beyond the scope of this paper. Moreover, β can be assumed as the leak term in the dynamics of leaky integrate and fire model due to its role in taking the value of the membrane potential away from the membrane threshold when there is no other inputs to the neuron. Inhibitory inputs in general can influence this parameter as well, however, for the sake of simplicity, we assumed it to be a constant. γ is a constant rate determining the number of spiking neurons at any given time interval *dt*. This term is proportional to the outgoing probability flux from the threshold θ in equation (11).

In comparison to the well-known Wilson-Cowan model (Wilson and Cowan, 1972), the ARM model assumes an exponential, instead of a sigmoidal, function as a transfer function for low input level. The reason is that in the balanced network, the firing rates of the neurons are low and therefore the activity of the populations are far from saturation. The ARM model can be considered as a special case of Wilson-Cowan model that was suggested for the dynamics of fluctuations around the mean firing rates of the populations for time-dependent inputs. The advantage of our model is that it can generate the statistics of the noise from the mean-field dynamics of the system in a self-consistent way.

### 3.3. State space analysis of self-generated noise

**Figure 5:**
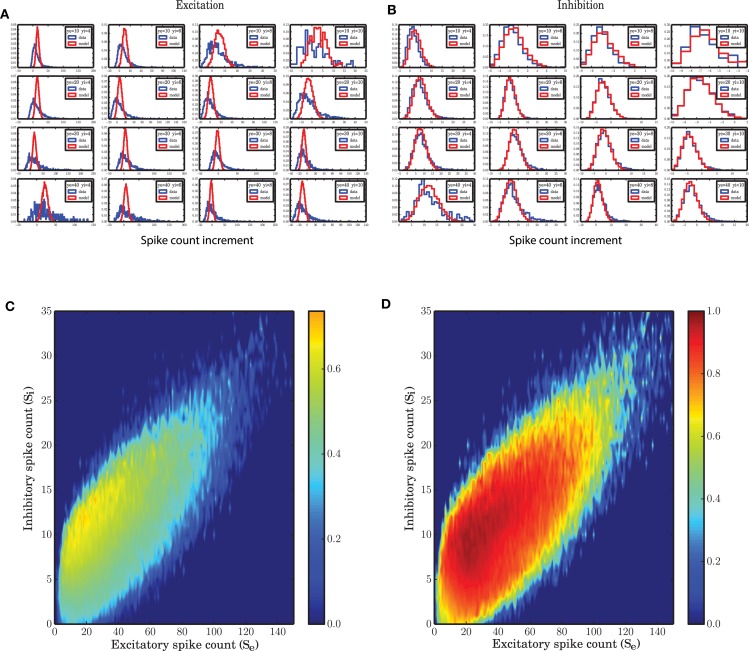
**State dependent distribution of the change (increments) in spike counts, (A)** for the excitatory population, **(B)** for the inhibitory population. Spiking network simulation (blue) compared with a realization of a model (red) show that the distribution of increments in spike counts are captured with a high accuracy for the inhibitory population. For the excitatory population the distributions of the increments in spike count in the model are symmetric. **(C,D)** Overlap between the distribution of the increments in the spiking network simulations and a stochastic implementation of the model for the **(C)** excitatory and **(D)** inhibitory activity.

**Figure 6:**
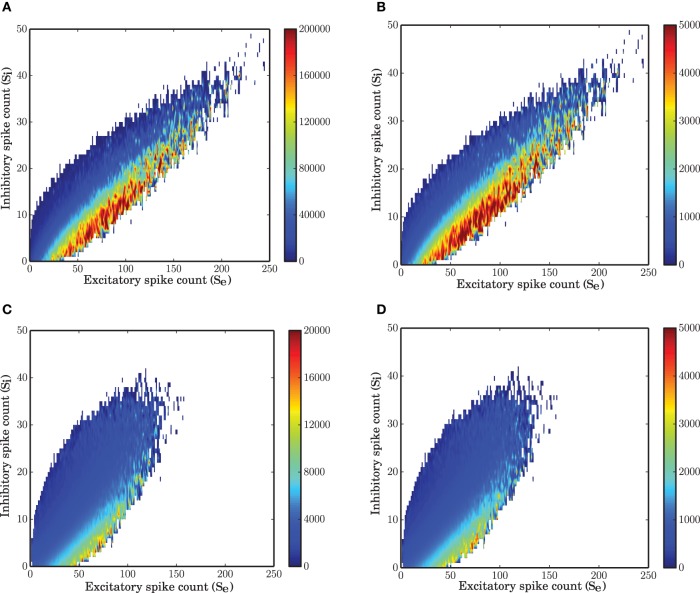
**State dependent variance of the derivatives of spike counts in small time bins of size 0.1 ms. (A)** Variance of the spike count derivatives for the excitatory population in a SNN. As the inhibitory spike counts decrease, the variance of increments increases. As a function of excitatory spike counts, the variance increases. **(B)** Variance of spike count derivatives for the inhibitory population in a SNN; the same pattern with a lower variance is observed in the case of inhibition. **(C)** Variance of spike count derivatives for the excitatory population in the stochastic implementation of the ARM model. The same pattern as **(A)** is observed but with a 10 times reduced variance. **(D)** Variance of spike count derivatives for the inhibitory population in the ARM model. The scale of the state dependent variance of noise is the same as in **(B)**.

Each pair of excitatory and inhibitory spike counts that are observed in the same time bin define a state in the state space. Spiking network simulations show that the future evolution of states during a particular trajectory of the system is highly dependent on the current state of the network. Therefore, the increment or decrement of the spike counts, as well as the derivative estimated from this (which are basically the difference between the spike counts at successive points in time divided the time interval 0.1 ms) are state dependent (Figures 5A,B). For each state, the distribution is heavy-tailed (positively skewed) and has a higher variance in excitation (compare the color-bars of Figures 6A,B). For a particular value of inhibitory spike counts, the variance increases as excitatory spike counts increase. Also, for a particular value of excitatory spike counts, increasing inhibitory spike counts results in less variability of the derivative of the spike counts for both excitatory and inhibitory populations. In other words, the more net excitation is in the system, the higher is the variance of the derivatives of the population spike counts. This statistical property of the network dynamics indicates that the noise model cannot be that of additive Gaussian white noise, as otherwise the variability in derivatives would be identical for the entire state space. The reason is that for any stochastic system governed by an equation of type $\dot{x}$ = *f*(*x*) + ξ, if the variance of $\dot{x}$ is the same for the entire state space, ξ could be considered as an additive white noise where the variance of the noise ξ is not state dependent. Otherwise, the noise term has to be state dependent, or non-additive. Multiplicatively interacting point processes are an explicit model for the interaction between neurons (Cardanobile and Rotter, 2010), with non-additive and non-Gaussian noise.

In the ARM model the transition probabilities are generally state dependent. Therefore, each state has a different distribution of the spike rate increments (derivatives). The model shows the same pattern of state dependent variance, however, the variance is not as high as that of spiking network simulations (Figures 6C,D). Particularly, for the excitatory spike counts the variance generated in the model is smaller by a factor of 10 (Figures 6A,C). However, this fact does not affect the distribution of the spike counts in the populations drastically (**Figure 9**) and the normalized correlation functions (**Figure 8**) are still recovered with a high accuracy.

Figure 5 shows that for some states in the state space, the distribution of the derivatives of the spike counts is reproduced by the ARM model with very high fidelity. Specifically for excitation, however, the model does not seem to generate enough variability in the excitatory spike counts. In Figures 5C,D the overlap between the distributions of the derivatives generated from SNN and ARM in the entire state space are represented by a number between 0, indicating no overlap, and 1, corresponding to a complete overlap, respectively. To calculate the overlap, first the state dependent distribution of the derivatives both for the model and the simulation data were normalized. Then the two distributions were compared, and for each possible value of the derivative, the minimum between the two distributions was determined. The integral over the minima is a number between 0 and 1 corresponding to the overlap. It is clear that the performance of the model in terms of variability of the inhibitory activity is very good (Figure 5D), however, the model is not as good in generating large enough variability in the derivative of the excitatory population. This might be related to the larger number of excitatory neurons compared to inhibitory ones, and therefore the bigger influence of the pairwise correlations among excitatory neurons that is ignored in the ARM model. The ARM model is based on the assumption that neurons, given the network is in a certain state, perform spike transition independently. This leads us to use binomial distributions to describe the transitions between the two neuronal states. This results in a linear scaling of the variance with the population size. As shown in Figures 6C,D (note the different scales of the color bars), the variance of the derivatives in both populations differ by a factor of 4, which is exactly the ratio of the excitatory and inhibitory population sizes. However, in the spiking network simulation, the variance of the derivatives for the excitatory population is much bigger than the inhibitory population (Figures 6A,B). We conclude that due to the linear relationship between the variance and the population size, the ARM model systematically underestimates the variance of the activity for the larger subpopulation in the network. This is a drawback of the model and in the discussion section we will suggest ways to overcome this problem. In the next section it is shown that the portion of the state space visited in a stochastic simulation of the model is less spread, and we attribute this fact to the reduced variability in the increments of the excitatory spike counts in the model.

### 3.4. Nonlinear isoclines

**Figure 7:**
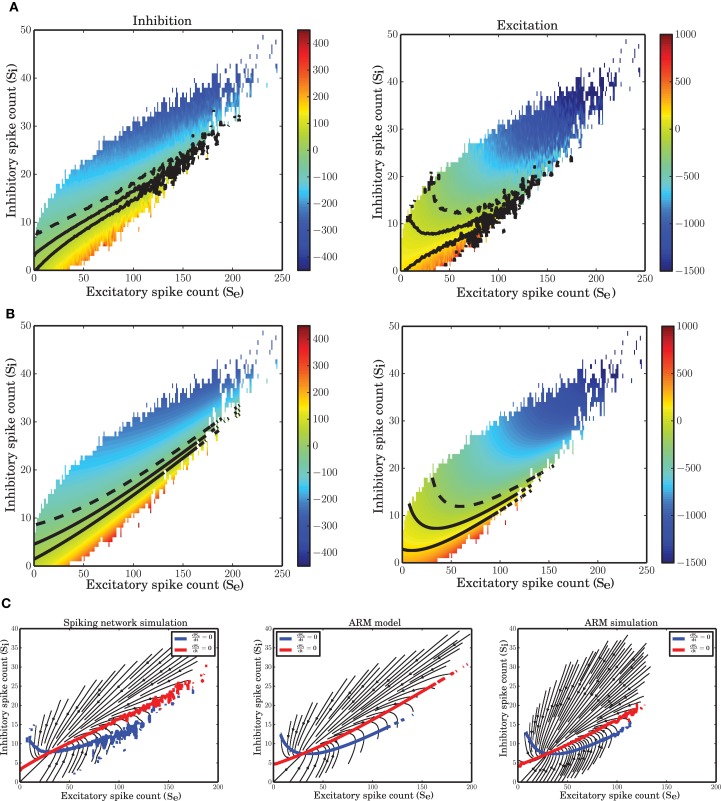
**Nonlinear isoclines and dynamic flow (vector-field) in the state space of excitatory and inhibitory spike counts. (A)** Left: Mean of the excitatory spike count derivatives; three levels of contour lines, −200, 0, 200 are shown in black. The isoclines are clearly nonlinear. Right: Mean of the inhibitory spike count derivatives; the contour lines are shown for −50, 0, 50. For inhibition, the nonlinearity is not dominant. **(B)** Mean of the derivatives of the excitatory and inhibitory spike counts in the ARM model. The isoclines are similar to those in a spiking network simulation. **(C)** Vector field extracted from a numerical simulation of spiking neuronal network (SNN; left) and simulation of the ARM Markov model (ARM); (middle: analytical model, right: stochastic simulation of the model). Parameters of the model were chosen such that the vector fields on the left and in the middle are identical. There is a good match between the vector fields and the nullclines in the simulation and in the model.

The state space of the system reconstructed from the simulated data (vector field shown in Figure 7) is a good representation of the mean-field dynamics that represents smooth transition of the average fluctuations toward the fixed point. Starting from an arbitrary initial condition in the state space, the mean-field dynamics leads the trajectory to a stable fixed point. However, due to recurrent activity and the finite size of the network, the trajectory driven by the mean-field is continuously perturbed and the result is a quasi-stochastic signal that only on average follows the mean-field dynamics. In other words, the vector field describes how a trajectory evolves on average.

The vector field has one component for each population representing the mean increment (or derivative) of the spike counts, given the two-dimensional state of the joint population. The excitatory and inhibitory components of the average state dependent derivatives are shown in Figure 7A. Isoclines in the state space represent contour lines of a particular value of the derivative. In Figure 7A, the isoclines corresponding to values −200, 0, 200 of the excitatory spike count derivative, and the −50, 0, 50 isoclines for the inhibitory spike count derivative are depicted in black. The inhibitory isoclines seem to be a linear function of excitatory and inhibitory spike counts. The isoclines for the derivatives of the excitatory spike counts are non-linear with a negative dependency of inhibition on excitation when there are only few excitatory spikes and relatively more inhibitory spikes are available. The slope of the dependency becomes positive when the number of excitatory spikes increases. The nullclines (0 isocline) of the system are the solutions of the two dimensional mean-field equations. An immediate conclusion from the shape of the nullclines is that the dynamics of a finite size network of excitatory and inhibitory neurons is nonlinear. More theoretical evidence for the nonlinearity of the dynamics, based on the system characteristic equation analysis is provided in the Supplementary Material.

The ARM model with parameters estimated form the vector field of the spiking network simulation reproduces the flow with very high fidelity (Figure 7C, middle). The stochastic implementation of the ARM model generates a comparable vector field with a similar shape of the nullclines (Figure 7C, right). However, the spread of the excitatory spike counts is reduced and therefore, compared to the spiking network simulation results, the variance of the excitatory spike counts is smaller (**Figure 9B**). The low variance of the excitatory spike counts may be explained by the state dependent distribution of the derivatives of the excitatory spike counts which the model generates (Figure 5A, red curve compared to the blue). In comparison with the spiking network simulation, the variance of this distribution is reduced. We conjecture that if the excitatory spikes are not only generated from a binomial distribution, but are also correlated with the recently generated inhibitory spike counts, the variance of this distribution will increase. Another way of generating excitatory spike counts from the occupation number would be to consider the two dimensional log-normal distribution of the spike counts in the state space.

### 3.5. Correlation functions of the two populations

**Figure 8:**
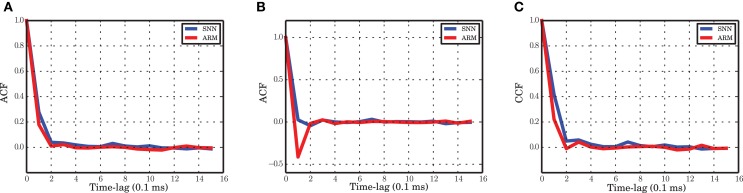
**Autocorrelation and crosscorrelation functions of the population activities. (A)** ACF for the excitatory population in spiking network simulation (blue) and ARM model (red). **(B)** ACF for the inhibitory population. **(C)** CCF between the excitatory and inhibitory populations. The labeling is the same as in **(A)**. The time-lag is in unites of 0.1 ms.

The balanced random network with stationary input is a highly recurrent system. A high correlation between excitation and inhibition nevertheless results in a decorrelated input to single neurons, because the recurrent inhibitory input cancels the effect of recurrent excitation (Renart et al., 2010). In an inhibition-dominated network, this provides an input to each neuron such that the mean membrane potential is below threshold and only fluctuations of the input cause threshold crossing and therefore result in a very low rate and irregular spiking activity of a neuron in the network. The high correlation between excitation and inhibition manifests itself in the cross-correlation between the spiking activity of the excitatory and inhibitory population (Figure 8C). The ARM model is capable of producing a high correlation function between excitation and inhibition, and the time scale of the correlation function is the same as for the network simulations.

The autocorrelation function of the excitatory and inhibitory population activities in the simulation of the spiking neuronal network has a very sharp decay in the range of the time scale selected for the Markov model (0.1 ms). For the excitatory population, the autocorrelation is close to zero after 0.2 ms and the ARM model reproduces the same correlation function (Figure 8A). We conclude that the model is able to capture the temporal dynamics of the excitatory population on such a short time scale. The autocorrelation of the inhibitory spiking activity decays faster and exhibits a small undershoot after 0.1 ms. The ARM model shows the same time-scale of correlation decay, however, the undershoot is more pronounced (Figure 8B). The shorter time scale of the inhibitory auto-correlation function compared to the excitatory one is also represented in the power spectral density of the excitatory and inhibitory populations, illustrated in Figures 1C,D. The excitatory population concentrates most of its power in a relatively narrow low frequency band compared to the inhibitory population.

### 3.6. Heavy tailed distribution of spike counts

**Figure 9:**
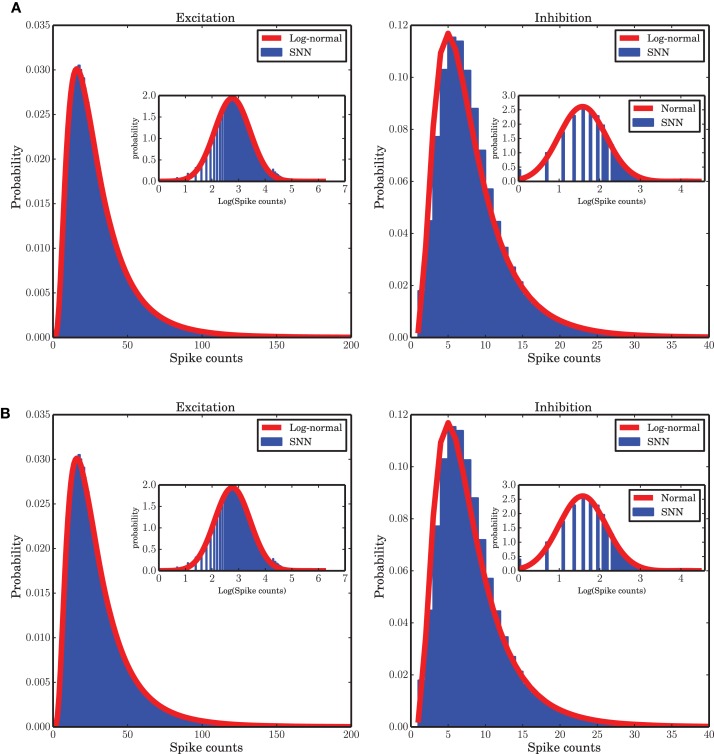
**Distribution of spike counts in short time bins (bin size 0.1 ms). (A)** For spiking network simulation, **(B)** for stochastic implementation of the ARM model. In both cases a log-normal fit (red curves) to the data (blue histograms) provides a good approximation. The insets in both panels show the distribution of the logarithm of the spike counts in blue and the maximum likelihood estimate of a log-normal distribution fit to the data on a log-scale.

Some previous studies of the temporal dynamics of interacting populations assume a Gaussian distribution of the activity around the fixed point solution of a low-dimensional system (Kriener et al., 2008; Tetzlaff et al., 2012; Helias et al., 2013), however, our numerical study shows that a log-normal distribution provides a good fit to the spike counts observed in the the spiking neuronal network (SNN) simulation (Figure 9A). Note that the population spike counts are bounded from above (due to the finite size of the system), a property that is not reflected by the log-normal distribution. Nevertheless, it was found to provide a good approximation for the range of data actually observed in our network simulations. Beyond the upper bound, the probability of spike counts is zero and the log-normal distribution is not valid. Therefore, strictly speaking, the distribution of such a bounded random variable is not heavy-tailed but we use this term for the positively skewed distribution of spike counts. The parameters μ and σ of the fitted log-normal distribution are 3.214, 0.664 and 1.938, 0.582 for the excitatory and inhibitory spike counts, respectively. The ARM model with the parameters extracted from the mean-field flow which was reconstructed from simulated data can capture this statistical property of the system (Figure 9B). The corresponding parameters of the ARM model are 3.405, 0.405 and 2.036, 0.539 for the excitatory and inhibitory spike counts, respectively. A log-normal distribution fits to the distribution of the spike counts generated in spiking network simulation and in the stochastic implementation of the ARM model very well, much better than possible alternative right-skewed distributions (gamma distribution, negative binomial distribution; data not shown). Insets of Figure 9 show a fitted normal distribution to the envelope of the distribution of the logarithm of the spike counts. The fact that spike counts are integer numbers needs to be taken into account in the fitting process. We conclude from these results that the spike counts of the population activity embedded in a network do not follow Poisson statistics, as the mean and the variance of the spike counts are not identical.

A log-normal distribution of activities in a different context, when there are inhomogeneous degree distributions or other quenched noise in the system, have been reported in different studies (Roxin et al., 2011; Mizuseki and Buzsáki, 2013; Buzsáki and Mizuseki, 2014). Outside the neuroscience literature it has been claimed that log-normality could be an emergent feature of competition among subgroups of individuals (Halloy, 1998; Halloy and Whigham, 2005). For a balanced random network with the same in-degree for all neurons, however, to the best of our knowledge, it is the first time that this statistical behavior is reported. We believe that this emergent feature is tightly related to the large-scale dynamics of the interacting excitatory and inhibitory populations. The state dependence of the transition rates, and the doubly stochastic nature of the Markov system that follows from it, might provide a formal explanation for this result.

### 3.7. Universality in balanced networks of different size

The network under study is a strongly connected network with identical in-degrees for all neurons. This implies that each neuron receives inputs from a fixed fraction of each population in the network. To keep the mean and the variance of the activity in the network limited, when the size *N* of the network tends to infinity, we scaled the synaptic weights by $\frac{1}{\sqrt{N}}$. We studied the network size effect on the variance of fluctuations and the nullclines of the reduced dimension system for 3 different networks of total size 7500, 12,500, and 20,000 neurons where in all these cases 20% of the neurons were inhibitory. The corresponding EPSP amplitude was 0.13, 0.1, and 0.08, respectively. To see whether there is any universal feature in the population dynamics of such networks, we normalized the spike counts of each population by the size of the population. This helps us to study dynamics of the fraction of the active neurons irrespective of the size of the network. To check the size invariance property of the ARM model, we rescaled equation (8) by the corresponding population size for the excitatory and inhibitory population. Introducing new variables $X_{e}=\frac{S_{e}}{N_{e}}$ and $X_{i}=\frac{S_{i}}{N_{i}}$, equation (8) could be rewritten in the following form

(12) $$\{\begin{array}{l} \begin{array}{l} {\dot{X}}_{e}(t)=\exp\left(c_{0}+{c^{\prime }}_{1}X_{e}(t)+{c^{\prime }}_{2}X_{i}(t)\right)\left(\gamma dt-X_{e}(t)\right)\\ \;-(\beta +\gamma )X_{e}(t)\\ {\dot{X}}_{i}(t)=\exp\left(c_{0}+{c^{\prime }}_{1}X_{e}(t)+{c^{\prime }}_{2}X_{i}(t)\right)\left(\gamma dt-X_{i}(t)\right)\\ \;-(\beta +\gamma )X_{i}(t) \end{array} \end{array}$$

**Figure 10:**
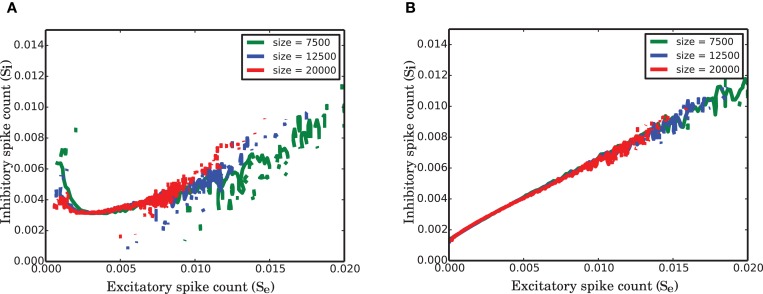
**Finite size effect on the nullclines of the normalized activity of the two populations in SNN. (A)** Null-cline for the excitatory population for networks of size 7 500, 12 500, 20 000. **(B)** Corresponding nullclines for the inhibitory population for the same network sizes as in **(A)**.

In equation (12), the coefficients *c*′_1_ and *c*′_2_ depend on the population sizes. The variables *X* are confined between 0 and 1 and comparing the dynamics of the networks with different sizes is reduced to a comparison between transition rates α, β, and γ. In our study, we were not able to identify an exact relationship between these parameters and the single neuron and connectivity parameters. However, Figure 10 shows that these parameters change such that the equation is invariant to network size.

As illustrated in Figure 10, the nullclines have the same shape irrespective of the size of the network, but the variability that is reflected in the total visited area of the state space decreases as the network size increases. The suggested active refractory model can reproduce the vector field of the spiking network simulation for different network sizes, provided that the parameters of the model are correctly estimated. Therefore, our conclusion is that the nonlinearity in the interaction between populations for stationary input and the heavy-tail (positively skewed) distribution of the activity are both universal properties of strongly connected networks of finite size.

### 3.8. Is the static nonlinear transfer function responsible for the nonlinear dynamics?

**Figure 11:**
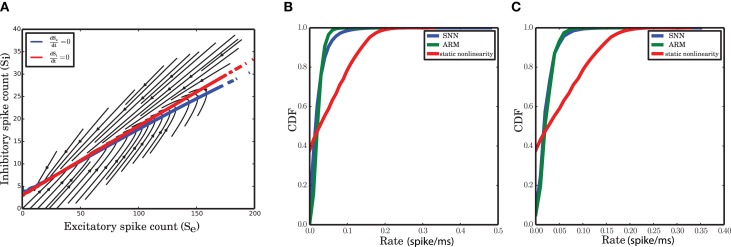
**Statistics and dynamics of equation (13). (A)** Flow of the two-dimensional system with almost linear null-clines. **(B,C)** CDF of the spike distributions for SNN, ARM and equation (13) for the inhibitory **(B)** and the excitatory **(C)** population.

Some studies of network dynamics assume that the stationary input-output transfer function of a neuron could provide a good description even for the time dependent activity of the network (Ledoux and Brunel, 2011; Pernice et al., 2012; Tetzlaff et al., 2012; Ostojic, 2014). The firing rate of a neuron in these models is typically approximated by the differential equation

(13) $$\tau {\dot{r}}_{i}(t)=-r_{i}(t)+F(r(t))$$

where **F** is the static nonliner function and **r** is the vector of the firing rate of other neurons in the network. This model, however, can be a good approximation for the temporal dynamics of the network only at large time scales, when the network activity is filtered over time and fluctuations have relatively low amplitude. Under these conditions, a linear approximation of the dynamics does provide a good match with the data. In our study, however, the time scale was chosen based on the time scale of the autocorrelation function of the network activity such that a wide range of frequencies contributes to the fluctuations. For *dt* = 0.1 ms, we demonstrate that the neural static nonlinearity cannot follow the delicate nonlinearity of the isoclines (Figure 11A) and the log-normality of the spike counts (Figures 11B,C). The nullclines of this model, after transforming the rates to spike counts, are almost linear functions of the respective spike counts (Figure 11A). Comparing the cumulative distributions of the spike counts generated from SNN, ARM and the input-output nonlinearity in equation (13) illustrates the discrepancy between the latter and the former. In fact, assuming that the instantaneous rates are equal to the stationary rates results in an overdispersed spike count distribution that does not match with that of SNN. Therefore, this assumption is obviously not correct for small time bins. These results show that system (13) is not valid on small time scales. On the other hand, as shown in the previous sections, the ARM model reproduces statistics and dynamics similar to the SNN data.

## 4. Discussion

In this study, we highlighted some properties of the large scale dynamics of finite-size balanced random networks in the inhibition dominated regime. It was shown that a linear system with additive Gaussian white noise cannot capture the statistics and dynamics of the two neuronal populations, and a more sophisticated model that represents the nonlinearity of the interactions and the statistics of the activity in a self-consistent way was suggested. We showed that a two state Markov process that the states of which reflect the coarse-grained membrane potential of a neuron, along with appropriate state-dependent transition probabilities, can reproduce the dynamics and statistics of a finite size network in the stationary balanced state in a satisfactory way. The state-dependent transition probabilities were all inferred from the numerical simulation of a spiking network of leaky integrate-and-fire neurons with stationary input. The approach was based on a reconstruction of the mean-field dynamics flow. The deterministic dynamics was complemented by a state-dependent stochastic component, assuming independent spiking in all neurons. This essentially leads to a binomial noise model, approximating Poisson statistics in very small time bins. On the level of the mean-field, the Active-Refractory Markov (ARM) model resembled the vector field extracted from the data with high fidelity. The population correlation functions of the spiking network simulation and the stochastic implementation of the model shared the same dynamical behavior.

We do not claim that the temporal dynamics of the finite size system is Markovian but a Markov model provides a good approximation. In general, the transition probabilities of the model depend on an unbounded history of the network activity (Cessac, 2011). However, we showed that a Markov model with a carefully chosen time scale is able to reflect some major statistical and dynamical properties of a balanced random network with fixed in-degrees. In the first part of the paper we explored the Markov properties of the network dynamics, and the size of time bins was chosen based on the similarity between the power spectra of a Markov model and the spiking network simulation. Then, we specifically considered a two-state Markov model for each neuron and estimated the state dependent transition probabilities from the simulated data. A self-consistent description that also accounts for the fluctuations of the finite-size system was obtained.

As outlined in detail in the Results section, there is a distinct similarity between the mean-field equations derived from the ARM model and the well-known Wilson-Cowan model (Wilson and Cowan, 1972) for the joint dynamics of excitatory and inhibitory neuronal populations. In the Wilson-Cowan model, the population response function, which gives the expected proportion of active neurons in a population as a function of the overall excitation in the system, derives from a cumulative unimodal density function and therefore typically has a sigmoidal shape. Using the Fokker-Planck equation to obtain the stationary distribution of the membrane potential and neglecting the absolute refractory time, a unimodal distribution will emerge. As discussed in this paper, the integral of the density function gives the transition probability from the refractory to the active state, the rate α in our model, as it reflects the local probability flux between the refractory and the active state. The local behavior of this function, for a wide range of the membrane potential between the threshold and reset, is well approximated by an exponential function and this approximation is quite good in comparison with spiking network simulations. A more precise result might come out if the CDF of the solution of equation (9) with free parameters is used for α. As shown in Figure 4, the CDF is a sigmoid function due to its unimodality. It is important to mention that the parameters of the model do not directly come from the CDF, but they should be estimated from the simulated data. An exponential function for the monotonically increasing CDF provides a good approximation for a wide range of network parameters and external input levels, as long as the network activity is asynchronous and irregular. Another comparison between the two models could be done on the kernel that is applied on the total excitation level in the network. In Wilson and Cowan (1972) an exponential kernel was used, which is justified by the impulse response of a leaky integrate and fire neuron.

The self-consistent noise model is an important feature of the model suggested in this article. The noise distribution is specific for each state, due to a deterministic dependency of the transition rates on the most recently generated spikes. Surprisingly, the state-dependent noise is such that it creates a strongly skewed distribution of the spike counts, similar to what is observed in the numerical simulations of spiking networks. The variance of the fluctuations generated by the model is, however, slightly smaller than the variance of the spike counts in the spiking network simulations. This is probably caused by the symmetric and relatively narrow state-dependent distribution of the noise in the excitatory population (Figure 5A). As mentioned in the result section, this problem is due to the assumption of independent spiking of individual neurons in each pool, implying the emergence of binomial distributions to describe the size of the neuronal populations that undergo a transition. As the variance of the outcome will be proportional to the total number of available neurons in each state, the variance of the increments will be directly proportional to the population size. One way of coping with this problem would be to create a correlation between the noise distributions in the two populations. Another way would be to consider the spike count distributions of the excitatory population given the spike counts of the inhibitory one. This way the heavy tail (positively skewed) distribution will come out automatically.

The nonlinear isoclines of the vector field provide strong evidence for a nonlinear interaction between the two neuronal populations. In particular the nullcline of the excitatory population exhibits very nonlinear behavior in the regime of small spike counts. Close to the fixed point of the flow, the excitatory nullcline changes direction. This property of the network can only be explained by a nonlinear model. It was already shown by Wilson and Cowan (1972) that different shapes of the nullclines emerge due to the asymmetry between excitation and inhibition, and because of their different signs in the argument of the population response function. In our model, however, the parameters of the two differential equations are the same, but different signs of the coefficients of the excitatory and inhibitory spike counts are enough to yield different shapes of the nullclines. A similar shape of the nullclines was in fact obtained in a model of the thalamo-cortical response transformations in the Barrel cortex of rodents (Pinto et al., 2003). We also checked whether the shape of the nullclines is invariant with regard to the size of the network. To compare the nullclines of networks of different sizes, however, it was necessary to normalize the activities of both populations. After normalization, the nullclines of networks with different sizes were almost identical, and it seems justified to claim that the nonlinear interaction is a universal property of strongly connected balanced networks. Since our method is based on the reconstruction of the dynamic flow, we conclude that our model can successfully capture the dynamics due to its size-invariance property.

It has been shown in van Vreeswijk and Sompolinsky (1996, 1998) that balanced random networks are extremely fast in following the temporal dynamics of an external input. This means among other things that the power spectral density of “spontaneous” network activity has a very broad frequency range and, as a result, the autocorrelation functions of the populations decay sharply. Our suggested model was successful in reproducing this fast temporal dynamics. Experimental evidence and theoretical studies suggest that dynamic responses of neocortical neurons are much faster when multiplicative input noise is imposed, compared to the case of additive noise (Lindner and Schimansky-Geier, 2001; Silberberg et al., 2004; Boucsein et al., 2009). We hypothesize that the multiplicative nature of the self-generated noise in balanced random network could also contribute to fast neuronal responses and sharp autocorrelation functions. Furthermore, an emergent property of balanced networks in the asynchronous irregular state is the high correlation between excitatory and inhibitory population activity, which was also successfully retrieved by our model. This is due to the fact that the same mean-field provides input to both populations. In other words, log(α) is a linear function of excitatory and inhibitory activity at any given point in time, with a positive weight for excitation and a negative weight for inhibition. This type of dependency makes a major contribution to correlating excitatory and inhibitory spike counts.

Recently, some studies have featured heavy-tail distributions of various phenomena in the brains of different species, supposedly a robust and important aspect of cortical computation (for a review see Buzsáki and Mizuseki, 2014), often approximated by log-normal distributions. Neurons in the auditory cortex (Deweese and Zador, 2008) and in the hippocampus and enthorinal cortex (Mizuseki and Buzsáki, 2013) of rats exhibit a log-normal distribution of firing rates. In Mizuseki and Buzsáki (2013) it is also shown that during the bursting activity state of the network, the fraction of all recorded neurons that fire a spike, for either stimulus-evoked or spontaneous activity, display a log-normal distribution. This, in turn, might be attributed to the log-normal distribution of the synaptic weights. Similarly, high density micro-electrode recordings of the human brain during sleep, in combination with a separation of excitatory and inhibitory cellular activities based on the spike wave-forms, has shown a log-normal distribution of firing rates (Peyrache et al., 2012). A theoretical study (Roxin et al., 2011) has shown that for a randomly connected network of excitatory and inhibitory LIF neurons with a random number of external inputs for each neuron and random synaptic efficacies, using an exponential *f*-*I* curve to describe the single neuron dynamics and a Gaussian distribution of inputs to each cell, a log-normal distribution of neural firing rates arises. In our study, however, all neurons had the same statistical inputs without any quenched variability, resulting in identical firing rates for all neurons in the network. However, the distribution of the spike counts for a large simulation time with small time bins was shown to be well fitted by a log-normal distribution. The suggested model was also able to represent this emergent statistical property of the network. This might emerge due to the exponential dependency of the transition probability α with suitably chosen parameters. In the theory of complex systems, the log-normal distribution is considered to be a universal statistical property of many natural systems (Halloy, 1998; Halloy and Whigham, 2005; Kobayashi et al., 2011). In particular, Kobayashi et al. (2011) showed that if the history of each component of the system defines its present state, log-normality becomes emergent. This could be tested on a network of multiplicatively interacting point processes that mimic the behavior of LIF neurons (Cardanobile and Rotter, 2010, 2011). However, a rigorous analysis shows that the Gibbs distribution is a unique invariant probability measure of the system under stationary input (Cessac, 2011). It might be interesting to investigate the underlying reason for the emergence of the log-normal distribution analytically.

We would like to stress once more the importance of choosing the right time scale for the ARM model. Due to the Markov assumption, the time bin must be chosen such that the influence of the past activity of the network on the transition rates (its memory) is minimal. This time scale is typically small, and we observe that a wide range of the power spectrum of the spiking activity is preserved. We conclude that the time dependent dynamics of the network is quite accurately captured. It was also shown in our paper that the typical input-output static nonlinearity of a neuron is neither suited to reproduce the nonlinear activity of the network, nor the log-normal distribution of the spike counts.

Markov models have been suggested before as models of the temporal dynamics of finite size networks (Soula and Chow, 2007; El Boustani and Destexhe, 2009; Cessac, 2011; Buice and Chow, 2013a). In Soula and Chow (2007), an approach similar to ours was proposed assuming a statistically homogeneous network of excitatory neurons. The difference to our work is that an active neuron is by definition a neuron that emits a spike, therefore only one transition probability from the silent to the active state is needed. This probability is proportional to the steady-state firing rate of the neuron. Also, a full-blown theoretical framework was introduced to calculate the first and second moment of fluctuations in the network. Our model, however, is different in the sense that the interaction between the excitatory and inhibitory population is taken into account, and that a high correlation between the activities of the two populations is preserved. Moreover, a heavy-tail distribution of the activity emerges as a result of the dynamical interaction between excitation and inhibition. However in Soula and Chow (2007) the distribution of the single population activity is symmetric due to the lack of inhibitory population. Our model on the other hand considers a neuron in the active state if its membrane potential is above some unspecified value of the membrane potential between threshold and rest. It takes the effect of leak into account by assuming a potential transition from the active to the refractory state without emitting a spike. El Boustani and Destexhe (2009) followed the same approach in continuous time for a sparse random network of excitatory and inhibitory neurons. Assuming quasi-stationarity, they derived the first two moment equations of the activity using the static input-output transfer function of a typical neuron in the network. However, Poisson statistics for each neuron was assumed and the transition function was calculated based on the mean activity of the network; therefore, the resultant distribution of the activity was Gaussian.

The main underlying assumption of the ARM model is the two-state Markovian single-neuron dynamics. For an appropriate choice of the time step, it was shown in the present study that the statistics and dynamics of recurrent networks of leaky integrate-and-fire neurons can be captured by the model. However, we neglected the role of absolute refractoriness in the dynamics of the membrane potential. Refractoriness shapes the low frequency range of the population dynamics (Mar et al., 1999; Spiridon and Gerstner, 1999). It could be modeled by introducing a chain of refractory states and thereby, increasing the dimensionality of the model (Toyoizumi et al., 2009). In the ARM model, absolute and relative refractoriness together, effectively, will have a wide distribution (for a relevant study, see Deger et al., 2010). Another aspect of our study is that synaptic transmission delays were neglected in the model as well as in our network simulations. In general, the transition probabilities depend on an unbounded past (Cessac, 2011) and delayed feedback makes the system non-Markovian (Vidybida and Kravchuk, 2013). It might be interesting to investigate whether a delayed α rate operating on the current pool of refractory neurons can represent the dynamics of the spiking network simulations. Furthermore, the important assumption of the ARM model is that all neurons statistically behave the same, because they all have the same in-degree. This allows us to reduce the dimensionality of the large scale dynamics and come up with a simple two state stochastic model of the system. If there is any inhomogeneity in the system, this model will not be a good candidate. Maybe for a network with homogeneous subpopulations, each component could be modeled by the ARM presented in this paper, with suitable parameters.

Population density methods are promising approaches for dimensionality reduction in dynamic networks. Including finite-size effects and correlations in the model, however, is a challenge. Deterministic density equations describing the temporal dynamics of finite-size networks were derived by using an eigenfunction expansion of the Fokker-Planck equation (Mattia and Del Giudice, 2002, 2004). Using a stochastic and deterministic approach, Buice and Chow recently suggested a mean-field equation and moment hierarchies of a density equation to obtain corrections arising from the finite size of the system and from correlations which are basically due to heterogeneities in the system (Buice and Chow, 2013a). For a homogeneous network, using the effective action approach of field theory (Buice and Chow, 2013c) and system size expansion around the mean-field density function (Buice and Chow, 2013b), they derived moment equations leading to the dynamics of mean and covariance in the network. Our suggestion for future exploration of the field is that a nonlinear set of Fokker-Planck equations for each population might be recast in the form of ARM model suggested in this paper. The more general model will have nonlinear and state dependent transition parameters that could be analytically derived and the stochastic behavior will emerge as a result of the finite size of the system.

Finally, to extend the model to cover the more general case of non-stationary and time dependent input, it is necessary to investigate the precise role of the external input in the ARM model. There are at least two possibilities: it could either be reflected in γ, or it could be included in the transition rate from the refractory to the active state, α. We suggest that the same data analysis method that we applied in this study might also help in determining the role of external time-dependent input in the model. Furthermore, it is possible to test whether this model can capture the dynamics of more than two interacting populations. However, this will be more challenging, as the dynamics of these type of networks are not necessarily stationary in time. Particularly, under certain conditions, switching dynamics between populations might arise (Litwin-Kumar and Doiron, 2012).

## Author contributions

Mathematical analysis and numerical experiments of this study were conceived and designed by Fereshteh Lagzi and Stefan Rotter. Numerical simulations and data analysis were performed by Fereshteh Lagzi, and supervised by Stefan Rotter. The paper was written by Fereshteh Lagzi and Stefan Rotter.

### Conflict of interest statement

The authors declare that the research was conducted in the absence of any commercial or financial relationships that could be construed as a potential conflict of interest.

## Figures and Tables

**Figure A1:**
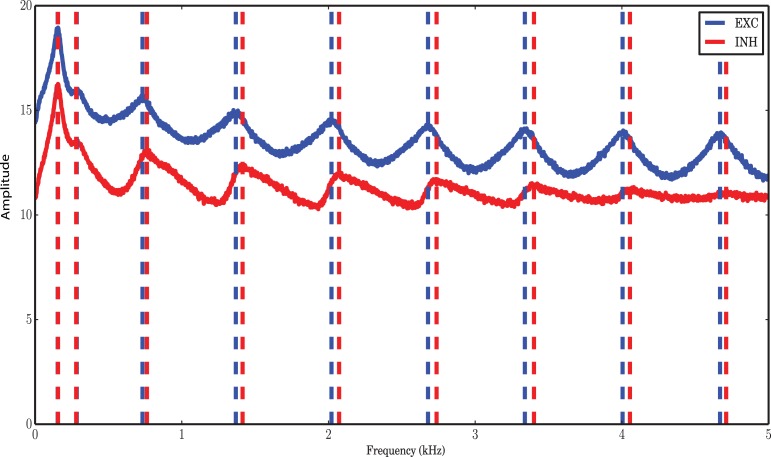
**Amplitude spectral density of spike counts with a bin size of 0.1 ms and *d* = 1.5 ms**. The entire spectrum of excitatory spike counts in bins of 0.1 ms is plotted in blue. The power spectrum of inhibitory spike counts under the same condition is plotted in red. Due to less number of neurons in the inhibitory population, its amplitude is lower than that of excitation. Dashed lines are plotted at frequencies corresponding to the peakes in the power spectra. For low frequencies, the peaks of the two spectra are the same. In the high frequency range the peaks in the amplitude spectrum of the inhibitory spike counts are slightly shifted with respect to those of excitatory ones.

## Appendix

### A.1. Properties of a linear system with Gaussian noise

In this section we highlight the main properties of a linear system and we will show that the temporal dynamics of finite size networks does not suggest a linear model. To demonstrate this, we consider a general two-dimensional linear system and will use two sources of Gaussian white noise to drive the excitatory and the inhibitory population. Then, we will discuss the features of this model due to the linear nature of the system and those which emerge due to the external drive. We assume in the following that *y_e_*(*t*) and *y_i_*(*t*) represent the instantaneous firing rates of the excitatory and inhibitory population. We consider an arbitrary communication delay in population interactions, which need not to be identical. Without loss of generality, we assume zero initial conditions for both populations. A coupled two-dimensional linear system in its general form is represented in the following way

(A1) $$\begin{array}{l} {\dot{y}}_{e}(t)=a_{ee}y_{e}(t-d)+a_{ei}y_{i}(t-d)+a_{e}I_{e}(t)\\ {\dot{y}}_{i}(t)=a_{ie}y_{e}(t-d)+a_{ii}y_{i}(t-d)+a_{i}I_{i}(t) \end{array}$$

Taking the Laplace transform of the above equation results in

(A2) $$\begin{array}{l} sY_{e}(s)=a_{ee}e^{-sd}Y_{e}(s)+a_{ei}e^{-sd}Y_{i}(s)+a_{e}I_{e}(s)\\ sY_{i}(s)=a_{ie}e^{-sd}Y_{e}(s)+a_{ii}e^{-sd}Y_{i}(s)+a_{i}I_{i}(s) \end{array}$$

which in matrix form is

(A3) $$\begin{array}{l} \left[\begin{array}{c} Y_{e}(s)\\ Y_{i}(s) \end{array}\right]={\left[\begin{array}{c} a_{ee}e^{-sd}-s\;a_{ei}e^{-sd}\\ \;a_{ie}e^{-sd}\;a_{ii}e^{-sd}-s \end{array}\right]}^{-1}\left[\begin{array}{c} -a_{e}I_{e}(s)\\ -a_{i}I_{i}(s) \end{array}\right]\\ \;=\frac{1}{G(s)}\left[\begin{array}{c} a_{ii}e^{-sd}-s\;-a_{ei}e^{-sd}\\ \;-a_{ie}e^{-sd}\;a_{ee}e^{-sd}-s \end{array}\right]\left[\begin{array}{c} -a_{e}I_{e}(s)\\ -a_{i}I_{i}(s) \end{array}\right]; \end{array}$$

where

$$G(s)=\left(a_{ii}e^{-sd}-s\right)\left(a_{ee}e^{-sd}-s\right)+a_{ie}a_{ei}e^{-2sd}$$

is called the characteristic equation of the linear system and appears in the denominator of both *Y_e_*(*s*) and *Y_i_*(*s*). The peaks in the power/amplitude spectra show the location of the zeros of the characteristic equation of the system. Therefore, the poles of the two components are identical and result in the same location of peaks in the power spectrum of *y_e_*(*t*) and *y_i_*(*t*). This statement is true in the more general case, when the inputs are different and non-Gaussian, since the two inputs play a role in shaping the power spectrum of both signals. The conclusion that we draw from this analysis is that if the power or amplitude spectra of two mutually interacting signals do not have the same peaks, then the interaction between them cannot be linear.

Equation (A3) shows that a coupled system as such behaves like a low-pass filter and for white noise input, the output would just be a filtered version of the noise. Since the distribution of the input is symmetric about its mean, the distribution of the output is also symmetric. Therefore, a non-symmetric distribution of the output must be interpreted as either a sign of nonlinearity of the system or the non-Gaussian nature of the input.

### A.2. Non-identical poles in the amplitude spectrum of excitatory and inhibitory spike counts

A balanced network of excitatory and inhibitory population, with the same characteristics of the network introduced in the Methods section, but with a synaptic delay of 1.5 ms and the total simulation time of 100 s, was simulated. The amplitude spectra of the excitatory and the inhibitory population are illustrated in Figure A1. In the low frequency regime of the dynamics, the peaks of the excitatory and inhibitory amplitude spectra are identical. However, for the high frequency part of the spectra the locations of the poles of the two populations are slightly shifted with respect to each other. This fact shows that the dynamics of the system that describe the temporal activity of the population interactions cannot be linear.
